# Supplementary material for: Mucoadhesive Buccal Film of Estradiol for Hormonal Replacement Therapy: Development and In-Vivo Performance Prediction
Source: Pharmaceutics. 2022 Feb 28;14(3):542. doi: 10.3390/pharmaceutics14030542 (PMC8955147; doi:10.3390/pharmaceutics14030542)
Supplement: Supplementary file 1 [file pharmaceutics-14-00542-s001.zip › pharmaceutics-1585793 suppl.pdf]

pharmaceutics

Article

Mucoadhesive Buccal Film of Estradiol for Hormonal Replacement Therapy: Development and In-Vivo Performance Prediction

SADIKALMAHDI ABDELLA[1,2], Franklin Afinjuomo [1], Yunmei Song [1], Richard Upton [3], and Sanjay Garg [1,*]

1 Pharmaceutical Innovation and Development (PIDG) Group, Clinical and Health Sciences, University of South Australia, Adelaide, SA 5000, Australia; sadikalmahdi.abdella@mymail.unisa.edu.au (S.A.); franklin.afinjuomo@unisa.edu.au (F.A.); may.song@unisa.edu.au (Y.S.)
2 School of Pharmacy, College of Health Sciences, Addis Ababa University, Zambia St, Addis Ababa 1000, Ethiopia
3 Clinical and Health Sciences, University of South Australia, Adelaide, SA 5000, Australia; richard.upton@unisa.edu.au
* Correspondence: sanjay.garg@unisa.edu.au

Table S1. Folding endurance of films prepared by co-solvency and nano-emulsion.

Formulation	Co-solvency	Nano-emulsion
F1	350	320
F2	325	333
F3	343	340
Mean	331	339

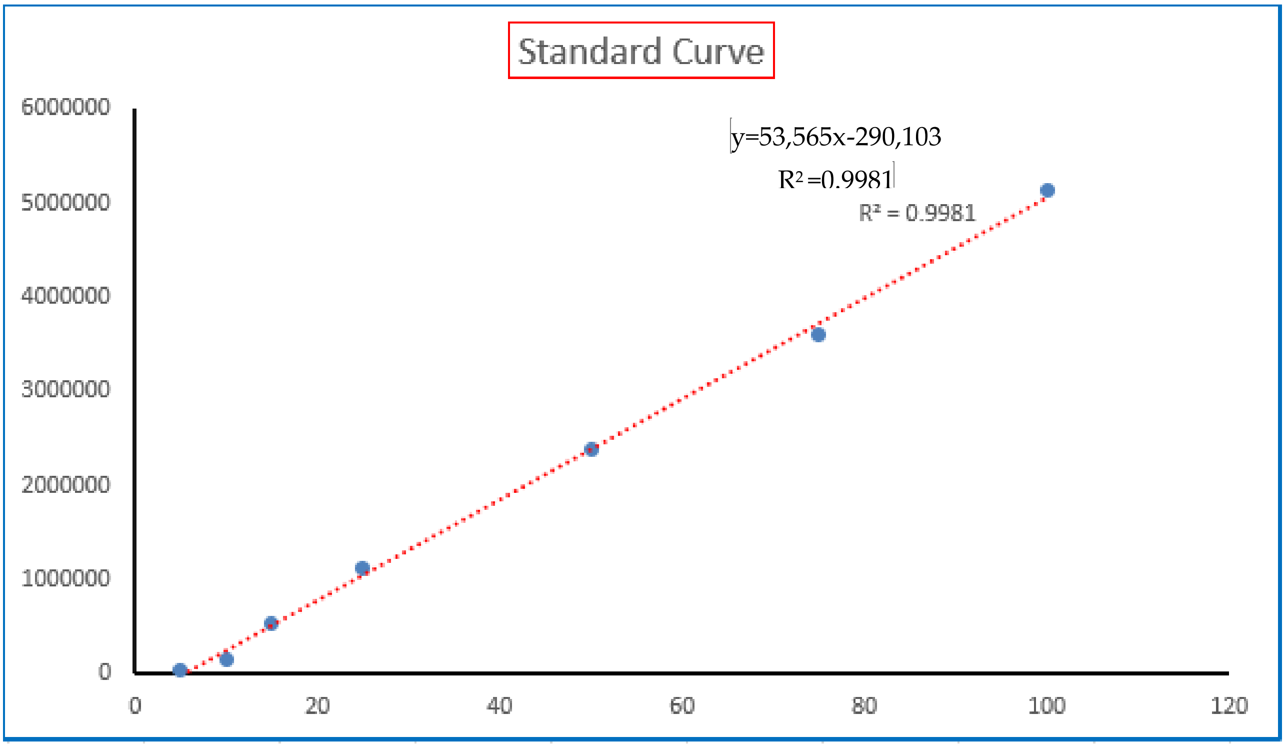

Figure S1. Calibration curve of estradiol and regression equation.
